# Immunoregulatory functions of mature CD10^+^ and immature CD10^–^ neutrophils in sepsis patients

**DOI:** 10.3389/fmed.2022.1100756

**Published:** 2023-01-04

**Authors:** Ming Liu, Guan Wang, Lin Wang, Yuqi Wang, Yuqing Bian, Hang Shi, Jie Liu

**Affiliations:** ^1^Department of Anesthesia, The Second Affiliated Hospital of Dalian Medical University, Dalian, China; ^2^Department of Dermatology, The First Affiliated Hospital of Dalian Medical University, Dalian, China

**Keywords:** sepsis, neutrophil, CD10, lymphocyte, immunoregulation

## Abstract

**Introduction:**

Neutrophil plays a more and more important role in sepsis with paralysis of immunoregulation. Till now, there was no biomarker to identify and isolate the mature and immature neutrophils in sepsis patients. CD10 shows on mature neutrophils at the latest stages of its differentiation. Our study aimed to investigate whether CD10 was a valid biomarker for distinguishing immature and mature neutrophil subgroups under septic conditions and their immunoregulatory effects on lymphocytes.

**Methods:**

Totally 80 healthy volunteers and 107 sepsis patients were recruited in this study. Fluorescence-conjugated anti-CD66b, and anti-CD10 monoclonal antibodies followed by incubation with specific anti-fluorochrome microbeads was used to isolate different subgroups of neutrophils. T cell apoptotic assays and T cell proliferation assays followed by flow cytometry analysis were used to evaluate the immunoregulatory effect of each subgroup of neutrophils.

**Results:**

(1) The cytological morphology of CD10^+^ neutrophils was mature and that of CD10^–^ neutrophils was immature in sepsis patients. (2) Mature CD10^+^ neutrophils inhibited the proliferation of T cell and immature CD10^–^ neutrophils promoted the T cell proliferation.

**Conclusion:**

(1) CD10 was a good biomarker to distinguish mature from immature neutrophils in sepsis patients. (2) Mature CD10^+^ and immature CD10^–^ neutrophils displayed opposite immunoregulatory effects on T cells in sepsis patients.

## 1. Introduction

Sepsis is a life-threatening disease caused by infection which leads to an imbalance in the immune response, with damage of tissue and organ (1–3). Sepsis is worldwide and seriously affects human health. According to the reports, the annual incidence of sepsis is 31 million people of the whole world (4, 5). Sepsis and its sequelae remain consistently high in all age groups, even under appropriate antibiotics and resuscitation, with the global mortality rate is as high as 17%, and sepsis is listed on the top 10 causes of death in the world (2). So far, the problem of sepsis and septic shock has not been overcome by medical doctors and scholars around the world, which consumes a large amount of medical manpower, material resources and financial cost (6). The United States spends nearly $20 billion per year on sepsis and septic shock (7).

Although the level of inflammatory mediators in sepsis patients is high, certain components of the immune system are strongly inhibited (8, 9). Some scholars have described sepsis as an immunosuppressive disease or immune paralysis (5, 8). In sepsis, lymphocyte dysfunction occurs, and the loss of lymphocyte function is associated with mortality in sepsis, other adverse outcomes, and reduced resistance to secondary infection. The mechanism of lymphocyte suppression in sepsis is not fully understood (8, 10).

In the past two decades, scholars have been studying more and more on the role of neutrophils in the regulation of immune responses. Many studies have found that sepsis can lead to neutrophil dysfunction at the site of infection and therefore to an imbalance of the immune response (11). They also found that in the blood of patients with autoimmune diseases (12–14) or cancers (15–17), there were different neutrophil-like subgroups that exhibit immunosuppressive or proinflammatory functions (8). Some of these neutrophil subgroups sink to the peripheral blood mononuclear cell layer after density gradient centrifugation of the peripheral blood, known as low density neutrophil (LDN). Immunosuppressive LDN is found in peripheral blood of patients with cancer, HIV-infected patients, and patients who have received the transplantation of organ (18, 19). Immunosuppressive neutrophil subgroups are also found in normal density neutrophil (NDN) (8, 12). In contrast, LDN in patients with autoimmune diseases such as systemic lupus erythematosus and psoriasis exhibit proinflammatory functions (8, 20, 21). But so far, no scholars have studied the immunoregulatory properties of LDN and NDN in sepsis patients.

A uniform and accurate description of the neutrophil isolation and identification method is the key to gaining a better understanding of neutrophil biological properties. Neutrophils in the pathological environment are composed of a mixed population of activated neutrophils and neutrophil heterogeneity at different stages of differentiation (17, 22). The expression of both CD11b and CD16 is often used to determine the maturation status of neutrophils, but it is not always reliable as they can vary variably upon activation or during neutrophil maturation (22–25). Therefore, it is necessary to identify more specific markers that allow accurate identification, rapid isolation, and functional characterization of mature neutrophils and detectable immature neutrophil subgroups in disease states.

CD10, also known as the common acute lymphoblastic leukemia antigen, specifically shows on mature neutrophils at the latest stages of its differentiation (8, 23–25). Therefore, this study will investigate whether CD10 can represent a valid marker for distinguishing mature neutrophil subgroups from immature neutrophil subgroups in LND and NDN under septic conditions; if so, we will continue to use CD10 to isolate and purify mature and immature neutrophils subgroups in sepsis patients’ LDN and NDN, and to study its immunomodulatory effects on lymphocytes.

## 2. Materials and methods

This was a prospective and controlled study. This study was approved by the ethics committee of the Second Hospital of Dalian Medical University and clinical trials registration number was ChiCTR-ROC-17013165.

### 2.1. Study participants

Totally 80 healthy volunteers and 121 sepsis patients were recruited from July, 2017 to February, 2020. All the sepsis patients were recruited from ICU, emergency ICU, emergency department, neurosurgery department, respiratory department, and gastrointestinal department.

#### 2.1.1. Sepsis patients

The definition of sepsis is infection + SOFA (sequential organ failure assess, Table 1) ≥2, and bacterial infection was confirmed by bacterial culture with corresponding bacteria, or by classic symptoms of infection such as erysipelas. The sources of infection were from blood, sputum, specimen from surgery, and urine tube. Non-bacterial infection was defined as negative results of blood culture or virus infection. Patients under 18 years old, with tumor or leukemia, immunosuppressive or immunopromotive treatment, history of organ transplantation, refusal of consent were excluded.

**TABLE 1:** Sequential organ failure assess.

System	Variable	0	1	2	3	4
Respiration	*P*aO_2_/*F*iO_2_ (mmHg)	>400	≤400	≤300	≤200	≤100
	Respiratory support				yes	yes
Coagulation	Platelets (10^9^/L)	>150	≤150	≤100	≤50	≤20
Liver	Bilirubin (μmol/L)	<20	20–32	33–101	102–204	>204
Cardiovascular	MAP	≥70	<70			
	Dopamine (ug/kg/min)			≤5 or	>5 or	>15 or
	Epinephrine (ug/kg/min)				≤0.1 or	>0.1 or
	Norepinephrine (ug/kg/min)				≤0.1 or	>0.1 or
	Dobutamine (yes/no)			Yes (any dose)		
Central nervous system	Glasgow coma score	15	13–14	10–12	6–9	<6
Renal	Creatinine (μmol/L)	<110	110–170	171–299	300–440	>440
	Urine output (ml)				201–500	<200

#### 2.1.2. Healthy volunteers

Totally 40 male and 40 female healthy volunteers were recruited.

##### 2.1.2.1. Inclusion criteria

(1) Age ≥ 18 years old;
(2) Body weight ≥ 50 kg, body mass index (BMI) between 19–26 kg/m^2^;
(3) Agreed to draw peripheral venous blood once during the whole study period, totally 2 ml;
(4) Subjects voluntarily signed the informed consent form;
(5) The subject held the physical examination report within 1 month.

##### 2.1.2.2. Exclusion criteria

(1) Suffering from serious cardiovascular and cerebrovascular diseases, kidney diseases, and liver diseases;
(2) Tumor;
(3) Patients with mental disorders;
(4) Drinking, smoking, and drug abuse;
(5) Take sedatives, analgesics, and psychotropic drugs for a long time;
(6) Pregnant and lactating women;
(7) Receive immunosuppressive or immunopromotive therapy.

### 2.2. Methods

#### 2.2.1. Cell isolation

##### 2.2.1.1. Isolation of lymphocytes

After diluting 2 ml of fresh anticoagulant blood of healthy volunteer with equal volume of phosphate buffered saline (PBS, TBD, China), carefully spread the diluted blood sample over the liquid level of 3 ml lymphocyte separating solution (TBD, China) in a 15 ml centrifuge tube. Then put the tube into the centrifuge tube with 400 *g* for 30–40 min at 20°C. After centrifugation, the cells in the centrifuge tube were divided into four layers from top to bottom: The first layer was the plasma layer, the second layer was the annular milky white lymphocytes, the third layer was the transparent separating liquid layer, and the fourth layer was the red cell layer. Carefully sucked the second layer of cells into another centrifuge tube, and mixed it with three times the volume of PBS solution, then centrifuged the mixture 100 *g* for 10 min, and discarded the supernatant. Resuspended the target cells with 0.5 ml of the corresponding liquid required for subsequent experiments.

##### 2.2.1.2. Isolation of CD66b^+^CD10^+/–^-LDN/NDN

Mononuclear cells and granulocytes were isolated by density gradient centrifugation (Ficoll–Paque, GE Healthcare Life Sciences). In a 15 ml centrifuge tube, added 2 ml peripheral blood sample from healthy volunteers/sepsis patients on the surface of 5 ml of human peripheral blood neutrophil separator (TBD, China). Then put them in the centrifuge for 30 min with 550 *g*. There were two layers of uniform milky white thin layers in the centrifuge tube. The upper layer was monocytes layer which contained LDN and the lower layer was NDN.

Isolation of CD66b^+^CD10^+/–^-LDN in previous upper layer and CD66b^+^CD10^+/–^-NDN in lower layer, respectively, were performed by incubation with fluorescence-conjugated anti-CD66b, and anti-CD10 monoclonal antibodies (mAbs), followed by incubation with specific anti-fluorochrome microbeads (Miltenyi Biotec, German) according to the protocol of manufactures. As determined by flow cytometry, Cell purity of all sorted populations was always 98%.

#### 2.2.2. Cell morphology

After purification by magnetic bead selection, CD66b^+^CD10^+/–^-neutrophil populations were stained by Reich Giemsa staining. Leica DFC 300FX Digital Color Camera on a Leica DM 6,000 B microscope at a 40× magnification was used to take picture. Both a pathologist and a hematologist, who were blinded to this study and cell samples, were assigned to identify the mature and immature neutrophil within at least 200 cells per slide of CD66b^+^CD10^+/–^-LDN/NDN.

The criteria of neutrophil morphology are as following: (1) Promyelocytes are characterized by a slightly often eccentrically indented nucleus in a blue cytoplasm; (2) myelocytes are characterized by a round or oval nucleus in a light blue cytoplasm; (3) metamyelocytes are characterized by a kidney-shaped nucleus in a pink cytoplasm; (4) band cells are smaller with a characteristic horseshoe-shaped nucleus of uniform thickness; and (5) mature neutrophils have a segmented nucleus typically characterized by 2–5 lobes separated by narrow filamentous bridge as illustrated in Figures 3B, 4B.

#### 2.2.3. Flow cytometry analysis

##### 2.2.3.1. Flow cytometry analysis for peripheral blood in healthy volunteers or sepsis patients

Added 100 μl peripheral anticoagulant blood in healthy volunteer or sepsis patient into the tube with following anti-human fluorochrome-conjugated mAbs or specific isotype controls: PE mouse anti-human CD66b (Biolegend, USA), PE-Cy7 mouse anti-human CD10 (Biolegend, USA), PE mouse IgG_1_κ isotype control (Biolegend, USA), PE-Cy7 mouse IgG_1_κ isotype control (Biolegend, USA), PerCP-vio700 mouse IgG_1_ Isotype (Miltenyi, German), and APC mouse IgG_1_κ isotype control (Biolegend, USA) 10 μl, respectively. After intubating for 15 min in the dark room at room temperature, 500 μl RBC Lysis Buffer (Macs, German) was added into the tube. The mixture was centrifuged with 2,000 rpm for 5 min after another intubating for 15 min in the dark room at room temperature, then removed the supernatant and added 500 μl PBS fluid into the precipitate to resuspend cells which was analyzed by eight-color three-laser-MACSQuant Analyzer (Miltenyi Biotec, German) while data analysis was performed by using FlowJo software (Tree Star, USA).

##### 2.2.3.2. Flow cytometry analysis for CD66b^+^CD10^+/–^-LDN/NDN in healthy volunteers or sepsis patients

After different neutrophil subgroups were obtained from step 2.2.1.2, typically 1 × 10^5^ cell/50 μl and the following anti-human fluorochrome-conjugated mAbs or specific isotype controls: CD66b (Biolegend, USA), PE-Cy7 mouse anti-human CD10 (Biolegend, USA), PerCP-vio700 mouse anti-human CD11b (Miltenyi, German), APC mouse anti-human CD16 (Biolegend, USA), PE mouse IgG_1_κ isotype control (Biolegend, USA), PE-Cy7 mouse IgG_1_κ isotype control (Biolegend, USA), PerCP-vio700 mouse IgG_1_ Isotype (Miltenyi, German), and APC mouse IgG_1_κ isotype control (Biolegend, USA) 10 μl, respectively were intubated for 15 min in the dark room at room temperature, 500 μl RBC Lysis Buffer (Macs, German) was added into the tube. The mixture was centrifuged with 2,000 rpm for 5 min after another intubating for 15 min in the dark room at room temperature, then removed the supernatant and added 500 μl PBS fluid into the precipitate to resuspend cells which was analyzed by eight-color three-laser-MACSQuant Analyzer (Miltenyi Biotec, German) while data analysis performed using FlowJo software (Tree Star, USA).

#### 2.2.4. Stimulation of neutrophils

Neutrophil populations of CD66b^+^CD10^+/–^-LDN/NDN in healthy volunteers or sepsis patients were suspended at 5 × 10^6^ cell/ml in RPMI 1,640 complete medium and pre-incubated with or without 2.5 ng/ml phorbol myristate acetate (PMA, Sigma, USA) for 30 min after purification by magnetic bead selection immediately.

#### 2.2.5. T cell apoptotic assays

T cell apoptotic assays were performed to qualify the apoptosis of T cell (CD4^+^ or CD8^+^) by flow cytometry to investigate the capacity of different neutrophil subgroups to regulate the apoptosis of T cell with Annexin V-FITC/PI Apoptosis Kit (Thermofisher, USA).

#### 2.2.6. T cell proliferation assays

CFSE dilution assays (CellTrace™ CFSE Cell Proliferation Kit, Life Technologies, USA) was performed to qualify the T cell (CD4^+^ or CD8^+^) proliferation by flow cytometry to investigate the capacity of neutrophils to suppress the responses of T cells which were pre-activated for 24 h with anti-CD3/CD28 mAbs, and then cultured in the absence or the presence of neutrophils for additional 72 h without specific T cell stimuli. The percentages of proliferating CD4^+^- or CD8^+^-T cells (expressed as CFSE- cells) were calculated from the total viable CD4^+^- or CD8^+^-T cells. All the T cells which were co-cultured with CD66b^+^CD10^+/–^-LDNs/NDNs neutrophil populations in sepsis patients or healthy volunteers were the same batch. The next steps were followed by the instruction of CellTrace™ CFSE Cell Proliferation Kit. Both neutrophils and T cells were cocultured in the 0.4 μm-pore size transwell (Corning, USA) with neutrophils on the top and T cells at the bottom of it.

### 2.3. Statistics

An unpaired two-tailed Mann–Whitney test (for comparison between two groups) or a 1-way ANOVA with Dunnett’s post-test (when multiple comparisons to control group were made) was used to perform the comparison of variables. *P*-values of less than 0.05 were considered significant and asterisks indicate significant increases: *P < 0.05; ^**^P ≤ 0.01; and ^***^P ≤ 0.001. GraphPad Prism Version 5 software (GraphPad Software, Inc., USA) was used to elaborating graphs. Flow chart was made by Flowjo V10.0.7 (Tree Star, USA).

## 3. Results

### 3.1. Patient characteristics

From July 2017 to December 2019, totally 80 healthy volunteers and 121 sepsis patients were recruited in this study. five patients’ families refused to participate the study, two sepsis patients withdraw the study after signing the informed consent form, and seven sepsis patients stopped treatment and left the hospital after taking blood samples. Therefore, totally 107 sepsis patients finally participated in the study.

Demographic characteristics of healthy volunteers and sepsis patients were listed in the Table 2.

**TABLE 2:** Demographic characteristics of healthy volunteers and sepsis patients.

	Sepsis patients	Healthy volunteers	*P*-value
Number	107	80	–
Male:female	49:58	40:40	0.8
Age (years)	43 (18–65)	49 (18–64)	0.3
BMI (kg/m^2^)	23.8 (20.1–25.9)	23.5 (19.9–25.8)	0.7
**Main diagnosis**			
Intestinal obstruction	24	–	–
Gastrointestinal perforation	21	–	–
Pancreatitis	17	–	–
Cholangiogenic shock	5	–	–
Pneumonia	21	–	–
Compound trauma	2	–	–
Myocardial infarction	14	–	–
Uremia	2	–	–
**Sites of infection**			
Abdominal cavity	72	–	–
Respiratory system	37	–	–
Hematogenous infection	1	–	–
Multiple infections	2	–	–
**Severity**			
SOFA	5 (2–10)	–	–

Data were shown as number (minimum, maximum).

### 3.2. Cell morphology of CD66b^+^CD10^+^-NDN in healthy volunteers and CD66b^+^CD10^+/–^-LDN/NDN in sepsis patients

The frequency of both CD66b^+^-LDN and CD66b^+^CD10^–^-NDN in healthy volunteers was rare, which was almost 0 [0.05% (0–0.1%)], so we could not get any cell in these two populations.

**FIGURE 1:**
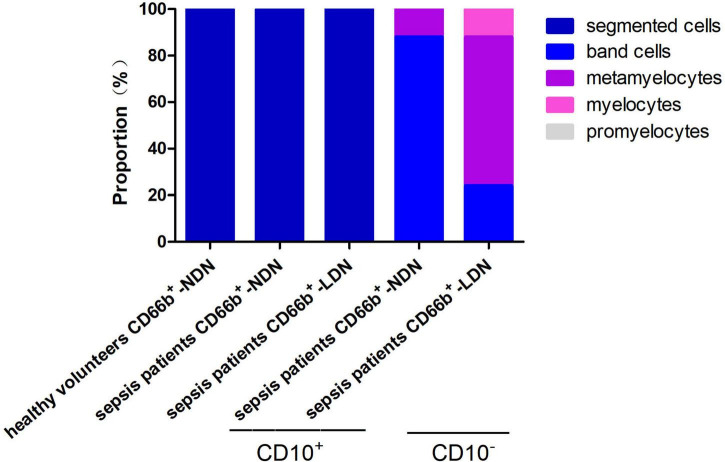
Cell morphology of CD66b^+^CD10^+^-NDN in healthy volunteers and CD66b^+^CD10^+^-LDN/NDN in sepsis patients.

In the populations of CD66b^+^CD10^+^-NDN in healthy volunteers, CD66b^+^CD10^+^-LDN/NDN in sepsis patients, the cell morphology was segmented cells with 2–4 lobulated nucleus, which were mature neutrophils. While in the population of CD66b^+^CD10^–^-LDN in sepsis patients, the cell morphology was myelocytes (24 ± 5%), metamyelocytes (64 ± 12%), and band cells (12 ± 6%); and the cell morphology was metamyelocytes (12 ± 4%) and band cells (88 ± 7%) in the population of CD66b^+^CD10^–^-NDN (Figure 1).

### 3.3. Frequency and proportion of CD66b^+^CD10^+^and CD66b^+^CD10^–^ in the peripheral blood of healthy volunteers and sepsis patients

**FIGURE 2:**
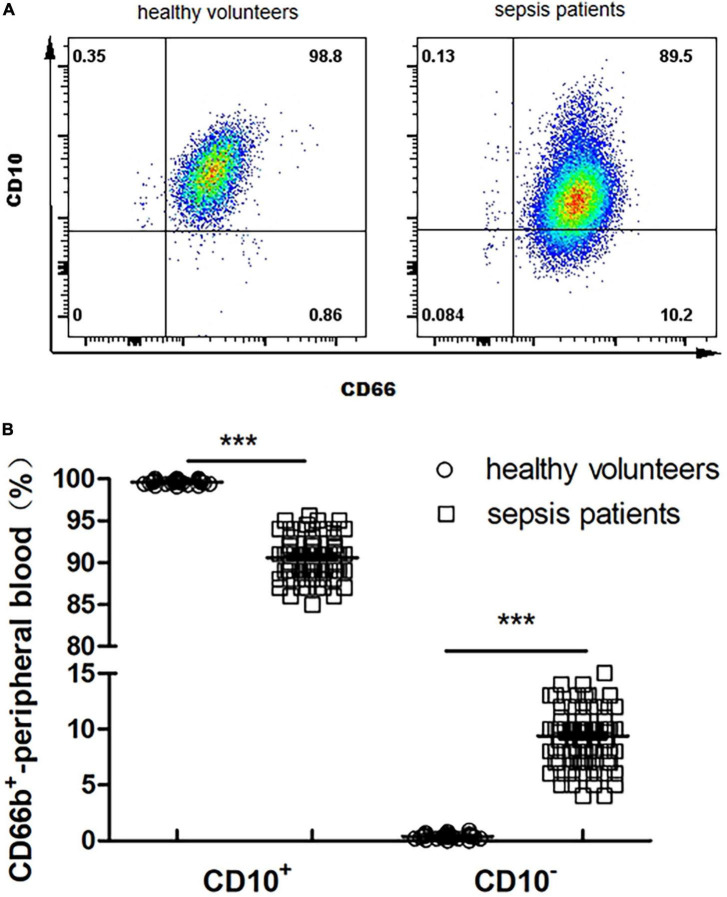
**(A)** Proportion of CD66b^+^CD10^+^ and CD66b^+^CD10^–^ of peripheral blood in healthy volunteers (80 cases) and sepsis patients (107 cases). Representative fluorescence-activated cell sorter (FACS) plots displaying CD66b and CD10 expression in peripheral blood in healthy volunteers. **(B)** Frequency of CD66b^+^CD10^+^ and CD66b^+^CD10^–^ of peripheral blood in healthy volunteers (80 cases) and sepsis patients (107 cases). Graph values indicate medians from independent experiments. Each symbol stands for a single healthy volunteer or sepsis patient. ***P < 0.001, by the Mann–Whitney *U*-test.

The proportion of CD66b^+^CD10^+^ in the peripheral blood of healthy volunteers is 99.6 ± 0.4%, and that of CD66b^+^CD10^–^ was 0.4 ± 0.2% (Figure 2A); The proportion of CD66b^+^CD10^+^ in the peripheral blood of sepsis patients was 90.2 ± 13.5%, and the proportion of CD66b^+^CD10^–^ was 10.3 ± 3.9% (Figure 2A), which was significantly different from that of healthy volunteers (*P* < 0.001) (Figure 2B).

**FIGURE 3:**
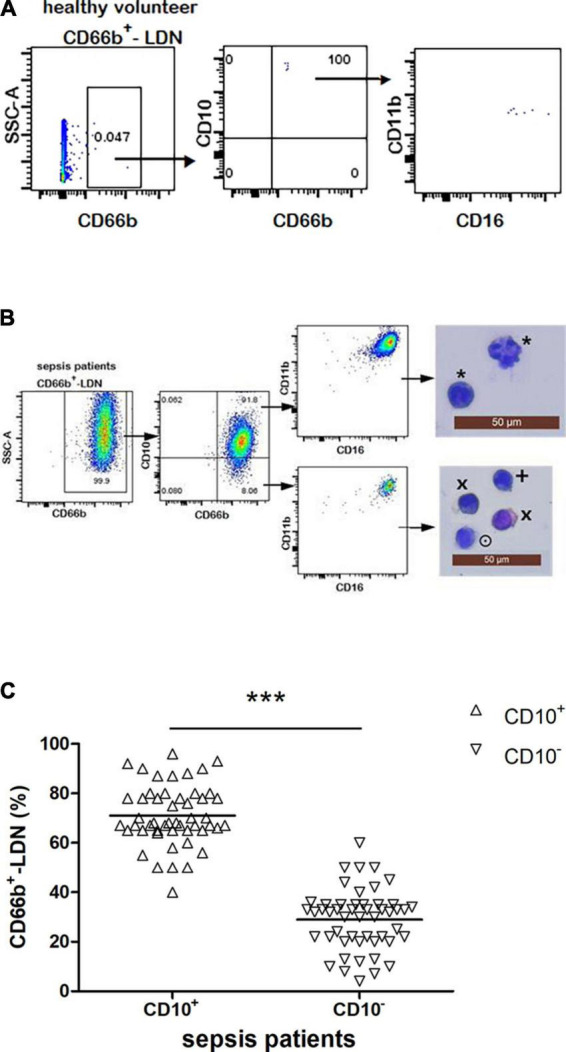
**(A)** Flow cytometry chart of CD66b^+^CD10^+^-LDN in healthy people. **(B)** Flow cytometry and cytological morphology of CD66b^+^CD10^+/–^-LDN in sepsis patients. *Represents the mature neutrophils with a segmented nucleus typically characterized by of 2–5 lobes; ^×^represents bend cell which were similar with a characteristics horseshoe-shaped nucleus of uniform thickness; ^+^represents metamyelocytes; ^⊙^represents myelocytes. **(C)** The frequency of CD66b^+^CD10^+^-LDN and CD66b^+^CD10^–^-LDN in sepsis patients with significant difference between these two groups (*P* < 0.001), ***P < 0.001 by Wilcoxon test.

**FIGURE 4:**
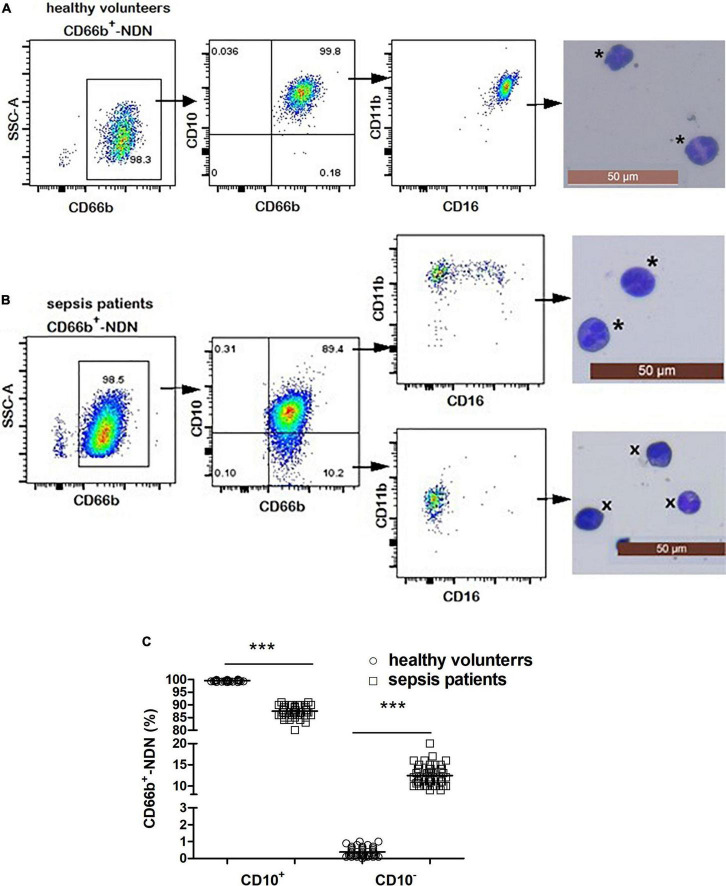
**(A)** Flow cytometry and cytological morphology of CD66b^+^CD10^+^-NDN in healthy volunteers. *Represents the mature neutrophils with a segmented nucleus typically characterized by of 2–5 lobes. **(B)** Flow cytometry and cytological morphology of CD66b^+^CD10^+/–^-NDN in sepsis patients. *Represents the mature neutrophils with a segmented nucleus typically characterized by of 2–5 lobes; ^×^represents bend cell which were similar with a characteristics horseshoe-shaped nucleus of uniform thickness. **(C)** The frequency of CD66b^+^CD10^+/–^-NDN in healthy volunteers and sepsis patients. Significant difference existed between CD66b^+^CD10^+^-NDN in healthy volunteers and sepsis patients, and CD66b^+^CD10^–^-NDN in healthy volunteers and sepsis patients (*P* < 0.001, respectively), ***P < 0.001 by Mann–Whitney *U*-test.

### 3.4. Frequency and phenotypic/morphologic characterization of CD66b^+^CD10^+/–^-LDN in healthy volunteers and sepsis patients

The frequency of CD66b^+^-LDN in healthy volunteers was rare, which was almost 0 [0.05% (0–0.1%)] (Figure 3A). The frequency of CD66b^+^-LDN in sepsis patients was 10.2 ± 3.4%, of which the frequency of CD66b^+^CD10^+^ was 62 ± 7% with the cell morphology was segmented cells and expression of CD16^bright^CD11b^bright^ (Figure 3B). The proportion of CD66b^+^CD10^–^ in CD66b^+^-LDN in sepsis patients was 38 ± 6%, with the cell morphology was myelocytes (24 ± 5%), metamyelocytes (64 ± 12%), and band cells (12 ± 6%), while the expression was also CD16^bright^CD11b^bright^ (Figure 3B). The difference between the frequency of CD66b^+^CD10^+^-LDN and CD66b^+^CD10^–^-LDN in sepsis patients was significant (*P* < 0.001) (Figure 3C).

### 3.5. Frequency and phenotypic/morphologic characterization of CD66b^+^CD10^+/–^-NDN in healthy volunteers and sepsis patients

The frequency of CD66b^+^CD10^+^-NDN in healthy volunteers was 99.9 ± 0.1% with segmented cells of 2–4 lobulated nucleus, which was mature neutrophils and expression of CD16 and CD11b was homogeneous, CD16^bright^CD11b^bright^; and the frequency of CD66b^+^CD10^–^-NDN was 0.1 ± 0.08% (Figure 4A).

The frequency of CD66b^+^CD10^+^-NDN in sepsis patients was 85 ± 8%, with the cell morphology of segmented cells and heterogeneous expression of CD16 and CD11b including CD16^bright^CD11b^bright^ and CD16*^dim^*CD11b^bright^; while the frequency of CD66b^+^CD10^–^-NDN was 13 ± 3%, with the cell morphology of metamyelocytes (12 ± 4%) and band cells (88 ± 7%) and heterogeneous expression of CD16 and CD11b including CD16*^dim^*CD11b^bright^ and CD16*^dim^*CD11b*^dim^* (Figure 4B).

The frequencies of CD66b^+^CD10^+^-NDN and CD66b^+^CD10^–^-NDN in healthy volunteers and sepsis patients were significantly different (*P* < 0.001) (Figure 4C).

### 3.6. CD66b^+^CD10^+^-NDN in healthy volunteers, CD66b^+^CD10^+/–^-NDN/LDN in sepsis patients did not inhibit or enhance lymphocyte apoptosis

**FIGURE 5:**
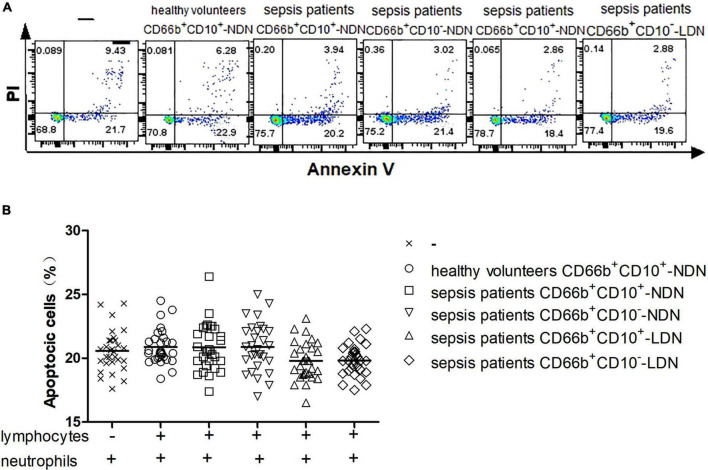
**(A)** Flow cytometry chart of the effect of different neutrophil subsets on lymphocyte apoptosis in healthy volunteers. **(B)** Comparison of the frequencies of apoptotic lymphocytes co-cultured with or without different neutrophil subsets. There was no significant difference among the six groups (*P* > 0.05).

After lymphocytes in healthy volunteers were co-cultured with or without different populations of neutrophils including CD66b^+^CD10^+^-NDN in healthy volunteers and CD66b^+^CD10^+/–^-LDN/NDN in sepsis patients for 24 h, flow cytometry was used to detect the proportion of lymphocytes apoptosis (Figure 5A). There was no statistical difference in the proportion of lymphocytes apoptosis among the six groups, *P* > 0.05 (Figure 5B).

### 3.7. The immunoregulatory properties of CD66b^+^CD10^+^-NDN in healthy volunteers and CD66b^+^CD10^+/–^-NDN/LDN in sepsis patients

**FIGURE 6:**
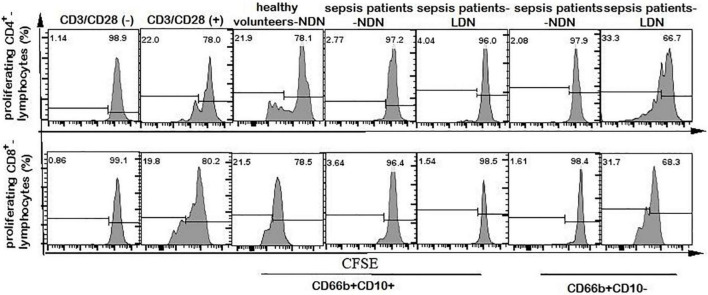
Flow cytometry chart of CD4^+^ and CD8^+^ lymphocyte proliferation. The first column of CD3/CD28 (–) represents the flow cytometry chart of lymphocyte proliferation without CD3/CD28 antibody stimulation, as a negative control. The second column of CD3/CD28 (+) represents the flow cytometry chart of lymphocyte proliferation stimulated by anti-CD3/CD28 antibody as a positive control. Columns 3–7 represent the flow cytometry chart of lymphocyte proliferation after stimulation by anti-CD3/CD28 and co-culturing with different neutrophils in 1:1 ratio for 72 h.

**FIGURE 7:**
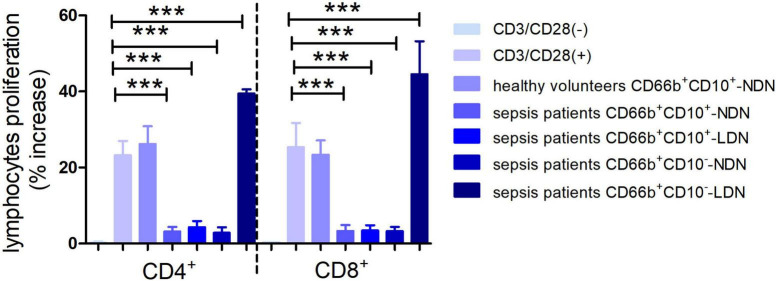
Histogram of the proliferation ratio of CD4^+^ and CD8^+^ lymphocytes. CD3/CD28 (–) represents the proportion of lymphocyte proliferation without stimulation by CD3/CD28 antibody as a negative control. CD3/CD28 (+) represents the proportion of lymphocyte proliferation stimulated by anti-CD3/CD28 antibody as a positive control. Columns 3–7 represent the lymphocyte proliferation ratio after stimulation by anti-CD3/CD28 and co-culturing with different neutrophils in 1:1 ratio for 72 h. ***P < 0.001.

Neutrophils of CD66b^+^CD10^+^-NDN in healthy volunteers had little effect on lymphocyte proliferation, and the percentage of CD4^+^ and CD8^+^ lymphocytes proliferation were 25.2 ± 3.7% and 26 ± 5.3%, respectively, with no significant difference when compared with the positive control group (23.2 ± 5.6% and 28 ± 6.4% respectively, *P* > 0.05), which was stimulated by CD3/CD28 (Figures 6, 7).

When co-cultured with neutrophils of CD66b^+^CD10^+/–^-NDN and CD66b^+^CD10^+^-LDN in sepsis patients, the percentages of CD4^+^ and CD8^+^ lymphocytes proliferation were significantly lower than that in control group (3.2 ± 1.4%, 4.3 ± 2.1%, 3.5 ± 1.8% and 2.8 ± 1.7%, 3.1 ± 2.0%, 3.9 ± 2.2%, respectively) (*P* < 0.001), which means CD66b^+^CD10^+/–^-NDN and CD66b^+^CD10^+^-LDN in sepsis patients inhibited lymphocyte proliferation. While the percentage of CD4^+^ and CD8^+^ lymphocytes proliferation when co-cultured with CD66b^+^CD10^–^- LDN in sepsis patients were much higher than that in positive controlled group (41.2 ± 6.7% and 48.7 ± 9.4%, respectively, *P* < 0.001), which means that CD66b^+^CD10^–^-LDN in sepsis patients could promote the proliferation of CD4 + and D8 + lymphocytes (Figures 6, 7).

## 4. Discussion

This was the first study to use CD10 to isolate and identify mature and immature neutrophils in LDN and NDN in sepsis patients, and also the first study to investigate the immunoregulatory function of mature and immature neutrophils in LDN and NDN in sepsis patients.

Previous studies showed that there was heterogeneity in the expression of CD antibody on the surface of neutrophils in the peripheral blood of sepsis patients and most previous studies used CD16 and CD11b to distinguish whether neutrophils were mature or not. Some researchers reported that in the case of cancer (20, 26), HIV-1 infection (27), pregnancy (28), and granulocyte colony-stimulating factor (G-CSF) (8), it might be difficult to distinguish mature neutrophils in immature ones by CD16 and CD11b and no scholar has studied the homogeneity and heterogeneity of NDN and LDN subgroups in sepsis patients (17, 22, 29–31). Marni et al. reported that CD10 was better than CD16 and CD11b in identifying and isolating mature neutrophils in G-CSF treatment donors with down-regulation of CD16 (8). The results of this study showed that in the LDN and NDN in sepsis patients, the cytological morphology of CD10^+^ was mature neutrophils (segmented cells), while in the LDN and NDN of sepsis patients, the cytological morphology of CD10^–^ was immature neutrophils. In this study, when we use CD16CD11b to distinguish whether neutrophils are mature or not, NDN subgroup in healthy people was homogeneous, while NDN and LDN subgroups in sepsis patients were heterogeneous. But when we combined the cell morphology of each subgroup, the results were quite different, even contradictory. As shown in Figure 3B of this study, the CD markers on the cell surface of the CD66b^+^CD10^+^-LDN and CD66b^+^CD10^–^-LDN subgroup in sepsis patients were both CD16^bright^CD11b^bright^, while the cell morphology of these two subgroups were different with mature neutrophils (segmented cells) in CD66b^+^CD10^+^-LDN and immature neutrophils (band cells, metamyelocytes, and myelocytes) in CD66b^+^CD10^–^-LDN group. Similar results were observed in the NDN subgroups of sepsis patients. Therefore, we believed that CD10 only expressed on mature neutrophils both in healthy volunteers and sepsis patients, and using CD16CD11b to distinguish mature and immature neutrophils in sepsis patients would cause errors. We concluded that CD10 was a good biomarker for identifying and separating mature and immature neutrophils in sepsis patients.

It has been proved that heterogeneous populations of immature and mature neutrophils coexist in the peripheral blood of patients with cancer, infection or autoimmune diseases, even in donors treated with G-CSF (8). The presence of immature granulocytes in peripheral blood provides important information for the enhancement of bone marrow activity. Such neutrophil heterogeneity results from systemic neutrophil activation and/or “emergency hematopoiesis,” which means the recruitment of bone marrow neutrophils increases, and apoptosis decreases. The same results were obtained in this study. There were no immature neutrophils in the peripheral blood and CD66b^+^-NDN in healthy people, while immature neutrophils appear in the peripheral blood, CD66b^+^-LDN/NDN in sepsis patients, and the proportion of immature neutrophils in sepsis patients was much higher than that in healthy volunteers. Our previous study proved that the frequency of CD66b^+^CD10^–^ in peripheral blood was a good biomarker to predict the bacterial infection in sepsis-suspected patients (32).

Under the circumstance of inflammation, the buoyancy properties of immature neutrophils might change and appear in LDN after blood density gradient centrifugation (33, 34). This was also consistent with the results of this study. In this study, the cytological morphology of CD66b^+^CD10^–^-NDN in sepsis patients was band cells (88 ± 7%) and metamyelocytes (12 ± 4%), while cytological morphology of CD66b^+^CD10^–^-LDN was band cells (12 ± 6%), metamyelocytes (64 ± 12%) and myelocytes (24 ± 5%), which means although the cytological phenotype of neutrophils in CD66b^+^CD10^–^-NDN and CD66b^+^CD10^–^-LDN were all immature, the left shift of nucleus was increased and led to the separating in the LDN after blood density gradient centrifugation.

Some studies reported that neutrophils might inhibit lymphocyte activity in some cases (35, 36). Wang et al. used PD-L1^+/–^ to differentiate neutrophils in septic mice and co-cultured a neutrophil subpopulation of PD-L1^+^ with monocytes in healthy mice, and the results showed that this neutrophil subpopulation increased lymphocyte apoptosis in healthy mice (37). While in our study, CD66b^+^CD10^+^-NDN in healthy volunteers, CD66b^+^CD10^+/–^-LDN/NDN in sepsis patients did not inhibit or enhance the apoptosis of lymphocytes. The difference between these results came from three aspects: First, the subjects were different. The subjects of our study were human, while Wang’s subjects were mice; Second, different markers were used for sorting neutrophil subsets. CD10, which can distinguish mature neutrophils from immature neutrophils, was used in this experiment, while PD-L1 was used in Wang’s experiment, which could not clarify the maturity of neutrophils; Third, the lymphocytes used in Wang’s study are monocytes + lymphocytes from the spleen of mice, while in our study lymphocytes I healthy volunteers were used. In the experiment of Marini et al. CD10 was used to isolate neutrophils from healthy human donors stimulated by G-CSF (8). It was found that the NDN of the donor and CD66b^+^CD10^+^-subgroup in LDN did not inhibit or enhance lymphocyte apoptosis in healthy people and the result was consistent with the results of ours.

In the T cell proliferation experiment, CD66b^+^CD10^+^-NDN in healthy volunteers had no effect on lymphocyte proliferation, CD66b^+^CD10^+/–^-NDN and CD66b^+^CD10^+^-LDN in sepsis patients inhibited lymphocyte proliferation; on the contrary, CD66b^+^CD10^–^-LDN in sepsis patients enhanced lymphocyte proliferation. Previous studies mostly focused on the neutrophils in NDN and few studies investigate the properties of neutrophils in LDN (38, 39), and results were consistent with ours, which was that the LDN and NDN of neutrophils would inhibit the proliferation of lymphocytes. But till now, there was no study was done to investigate the immunoregulatory function of mature and immature neutrophils of LDN and NDN. In our study, we found that immature CD66b^+^CD10^–^-LDN in sepsis patients showed the opposite behavior which was promoting the proliferation of lymphocytes. This result was consistent with that of Marini’s study (8).

In the study of Marini et al. the immature CD66b^+^CD10^–^-LDN in healthy human donors stimulated by G-CSF showed promotion of lymphocytes proliferation when co-cultured with lymphocytes at a ratio of 5:1, and the ration in our study was 1:1. Therefore, we supposed to conclude that the relative proportion of mature and immature LDN in sepsis patients determines the final type of immune regulation. This result might remind the clinicians that the paralysis of immunoregulation was not the only situation that existed in sepsis patients anymore, and treatment should be adjusted according to the final immunoregulatory function in the sepsis patients.

As immature neutrophils, CD66b^+^CD10^–^-NDN and CD66b^+^CD10^–^-LDN played opposite immunoregulatory function. This result was considered to be related to the different cell composition of the two subsets. CD66b^+^CD10^–^-LDN in sepsis patients was mainly composed of metamyelocytes and myelocytes, while the CD66b^+^CD10^–^-NDN subgroup in sepsis patients was mainly composed of band cells. Pillay et al. showed that CD16*^dim^*CD62L^bright^ band cells could not affect T cell proliferation (35), but Guerin et al. reported that CD14^–^CD24^+^ immature neutrophils (possibly band cells) isolated from LDNs of sepsis patients showed cytotoxicity to lymphocytes (36), while Singhal et al. recently reported that the CD66b^+^CD10^–^ band cells derived from bone marrow might produce promoting proliferation of lymphocytes (40). All these controversial results might be explained by the different methods of neutrophil isolation or the different composition of the immature neutrophil population. Therefore, it is necessary to define the specific immunomodulatory properties of band cells and other immature neutrophils.

From the results we got in our study, we concluded that: (1) CD10 is a good biomarker to distinguish mature and immature neutrophils in sepsis patients. (2) CD66b^+^CD10^+^-NDN in healthy volunteers, CD66b^+^CD10^+/–^-NDN/LDN in septic patients had no inhibitory or potentiating effects on lymphocyte apoptosis. (3) CD66b^+^CD10^+/–^-NDN and CD66b^+^CD10^+^-LDN in sepsis patients inhibited lymphocyte proliferation, while in contrast, CD66b^+^CD10^–^-LDN from sepsis patients promotes lymphocyte proliferation.

In the future study, we would investigate the mechanism of opposite immunoregulatory function these different neutrophil subgroups in sepsis patients.

## Data availability statement

The original contributions presented in this study are included in the article/supplementary material, further inquiries can be directed to the corresponding authors.

## Ethics statement

The studies involving human participants were reviewed and approved by the Ethics Committee of the Second Hospital of Dalian Medical University. The patients/participants provided their written informed consent to participate in this study. Written informed consent was obtained from the individual(s) for the publication of any potentially identifiable images or data included in this article.

## Author contributions

All authors listed have made a substantial, direct, and intellectual contribution to the work, and approved it for publication.

## References

[1] Napolitano L. Sepsis 2018: definitions and guideline changes. *Surg Infect.* (2018) 19:117–25. 10.1089/sur.2017.278 29447109

[2] Oczkowski S Alshamsi F Belley-Cote E Centofanti J Hylander Møller M Nunnaly M Surviving sepsis campaign guidelines 2021: highlights for the practicing clinician. *Pol Arch Intern Med.* (2022) 132:16290. 10.20452/pamw.16290 35791800

[3] Stephen A Montoya R Aluisio A. Sepsis and septic shock in low- and middle-income countries. *Surg Infect.* (2020) 21:571–8. 10.1089/sur.2020.047 32401160

[4] Rello J Valenzuela-Sanchez F Ruiz-Rodriguez M Moyano S. Sepsis: a review of advances in management. *Adv Ther.* (2017) 34:2393–411. 10.1007/s12325-017-0622-8 29022217 PMC5702377

[5] Hunt A. Sepsis: an overview of the signs, symptoms, diagnosis, treatment and pathophysiology. *Emerg Nurse.* (2019) 27:32–41. 10.7748/en.2019.e1926 31475503

[6] Mirijello A Tosoni A On Behalf Of The Internal Medicine Sepsis Study Group. New strategies for treatment of sepsis. *Medicina.* (2020) 56:527. 10.3390/medicina56100527 33050538 PMC7599752

[7] Jain S. Sepsis: an update on current practices in diagnosis and management. *Am J Med Sci.* (2018) 356:277–86. 10.1016/j.amjms.2018.06.012 30286823

[8] Marini O Costa S Bevilacqua D Calzetti F Tamassia N Spina C Mature CD10(+) and immature CD10(–) neutrophils present in G-CSF-treated donors display opposite effects on T cells. *Blood.* (2017) 129:1343–56. 10.1182/blood-2016-04-713206 28053192

[9] Salomao R Ferreira B Salomao M Santos S Azevedo L Brunialti M. Sepsis: evolving concepts and challenges. *Braz J Med Biol Res.* (2019) 52:e8595. 10.1590/1414-431x20198595 30994733 PMC6472937

[10] Jacobi J. The pathophysiology of sepsis-2021 update: part 1, immunology and coagulopathy leading to endothelial injury. *Am J Health Syst Pharm.* (2021) 79:329–37. 10.1093/ajhp/zxab380 34605875 PMC8500113

[11] Demaret J Venet F Friggeri A Cazalis M Plassais J Jallades L Marked alterations of neutrophil functions during sepsis-induced immunosuppression. *J Leukoc Biol.* (2015) 98:1081–90. 10.1189/jlb.4A0415-168RR 26224052

[12] Bowers N Helton E Huijbregts R Goepfert P Heath S Hel Z. Immune suppression by neutrophils in HIV-1 infection: role of PD-L1/PD-1 pathway. *PLoS Pathog.* (2014) 10:e1003993. 10.1371/journal.ppat.1003993 24626392 PMC3953441

[13] Cloke T Munder M Bergin P Herath S Modolell M Taylor G Phenotypic alteration of neutrophils in the blood of HIV seropositive patients. *PLoS One.* (2013) 8:e72034. 10.1371/journal.pone.0072034 24039734 PMC3767740

[14] Rieber N Wecker I Neri D Fuchs K Schäfer I Brand A Extracorporeal photopheresis increases neutrophilic myeloid-derived suppressor cells in patients with GvHD. *Bone Marrow Transplant.* (2014) 49:545–52. 10.1038/bmt.2013.236 24464140

[15] Solito S Marigo I Pinton L Damuzzo V Mandruzzato S Bronte V. Myeloid-derived suppressor cell heterogeneity in human cancers. *Ann N Y Acad Sci.* (2014) 1319:47–65. 10.1111/nyas.12469 24965257

[16] Talmadge J Gabrilovich D. History of myeloid-derived suppressor cells. *Nat Rev Cancer.* (2013) 13:739–52. 10.1038/nrc3581 24060865 PMC4358792

[17] Moses K Brandau S. Human neutrophils: their role in cancer and relation to myeloid-derived suppressor cells. *Semin Immunol.* (2016) 28:187–96. 27067179 10.1016/j.smim.2016.03.018

[18] Tay S Celhar T Fairhurst A. Low-density neutrophils in systemic lupus erythematosus. *Arthritis Rheumatol.* (2020) 72:1587–95. 10.1002/art.41395 32524751 PMC7590095

[19] Matthews N Burton C Alfred A. Low-density neutrophils in chronic graft versus host disease (cGVHD) are primarily immature CD10(–) and enhance T cell activation. *Clin Exp Immunol.* (2021) 205:257–73. 10.1111/cei.13612 33932293 PMC8274189

[20] Choi J Suh B Ahn Y Kim T Lee J Lee S CD15+/CD16low human granulocytes from terminal cancer patients: granulocytic myeloid-derived suppressor cells that have suppressive function. *Tumour Biol.* (2012) 33:121–9. 10.1007/s13277-011-0254-6 22081309

[21] Tsuda Y Fukui H Asai A Fukunishi S Miyaji K Fujiwara S An immunosuppressive subtype of neutrophils identified in patients with hepatocellular carcinoma. *J Clin Biochem Nutr.* (2012) 51:204–12. 10.3164/jcbn.12-32 23170048 PMC3491245

[22] Dumitru C Moses K Trellakis S Lang S Brandau S. Neutrophils and granulocytic myeloid-derived suppressor cells: immunophenotyping, cell biology and clinical relevance in human oncology. *Cancer Immunol Immunother.* (2012) 61:1155–67. 10.1007/s00262-012-1294-5 22692756 PMC11028504

[23] Elghetany M. Surface antigen changes during normal neutrophilic development: a critical review. *Blood Cells Mol Dis.* (2002) 28:260–74. 10.1006/bcmd.2002.0513 12064921

[24] Cossman J Neckers L Leonard W Greene W. Polymorphonuclear neutrophils express the common acute lymphoblastic leukemia antigen. *J Exp Med.* (1983) 157:1064–9. 10.1084/jem.157.3.1064 6220104 PMC2186955

[25] McCormack R Nelson R LeBien T. Structure/function studies of the common acute lymphoblastic leukemia antigen (CALLA/CD10) expressed on human neutrophils. *J Immunol.* (1986) 137:1075–82. 2941484

[26] Rodriguez P Ernstoff M Hernandez C Atkins M Zabaleta J Sierra R Arginase I-producing myeloid-derived suppressor cells in renal cell carcinoma are a subpopulation of activated granulocytes. *Cancer Res.* (2009) 69:1553–60. 10.1158/0008-5472.CAN-08-1921 19201693 PMC2900845

[27] Cloke T Munder M Taylor G Müller I Kropf P. Characterization of a novel population of low-density granulocytes associated with disease severity in HIV-1 infection. *PLoS One.* (2012) 7:e48939. 10.1371/journal.pone.0048939 23152825 PMC3496742

[28] Ssemaganda A Kindinger L Bergin P Nielsen L Mpendo J Ssetaala A Characterization of neutrophil subsets in healthy human pregnancies. *PLoS One.* (2014) 9:e85696. 10.1371/journal.pone.0085696 24551035 PMC3923728

[29] Carmona-Rivera C Kaplan M. Low-density granulocytes: a distinct class of neutrophils in systemic autoimmunity. *Semin Immunopathol.* (2013) 35:455–63. 10.1007/s00281-013-0375-7 23553215 PMC4007274

[30] Pedersen C Borup R Fischer-Nielsen A Mora-Jensen H Fossum A Cowland J Changes in gene expression during G-CSF-induced emergency granulopoiesis in humans. *J Immunol.* (2016) 197:1989–99. 10.4049/jimmunol.1502690 27481851

[31] Hubl W Andert S Thum G Ortner S Bayer P. Value of neutrophil CD16 expression for detection of left shift and acute-phase response. *Am J Clin Pathol.* (1997) 107:187–96. 10.1093/ajcp/107.2.187 9024067

[32] Liu J Shi H Yu J Xiong J. CD10 is a good biomarker to predict bacterial infection in sepsis-suspected patients. *Acta Med Mediterr.* (2019) 35:2851.

[33] Manz M Boettcher S. Emergency granulopoiesis. *Nat Rev Immunol.* (2014) 14:302–14. 10.1038/nri3660 24751955

[34] Orr Y Taylor J Bannon P Geczy C Kritharides L. Circulating CD10-/CD16low neutrophils provide a quantitative index of active bone marrow neutrophil release. *Br J Haematol.* (2005) 131:508–19. 10.1111/j.1365-2141.2005.05794.x 16281943

[35] Pillay J Kamp V van Hoffen E Visser T Tak T Lammers J A subset of neutrophils in human systemic inflammation inhibits T cell responses through Mac-1. *J Clin Invest.* (2012) 122:327–36. 10.1172/JCI57990 22156198 PMC3248287

[36] Guerin E Orabona M Raquil M Giraudeau B Bellier R Gibot S Circulating immature granulocytes with T-cell killing functions predict sepsis deterioration*. *Crit Care Med.* (2014) 42:2007–18. 10.1097/CCM.0000000000000344 24942511

[37] Wang J Li J Zhao Y Yi W Bian J Wan X Up-regulation of programmed cell death 1 ligand 1 on neutrophils may be involved in sepsis-induced immunosuppression: an animal study and a prospective case-control study. *Anesthesiology.* (2015) 122:852–63. 10.1097/ALN.0000000000000525 25437496

[38] Arens C Bajwa S Koch C Siegler B Schneck E Hecker A. Sepsis-induced long-term immune paralysis–results of a descriptive, explorative study. *Crit Care.* (2016) 20:93. 10.1186/s13054-016-1233-5 27056672 PMC4823837

[39] Monneret G Venet F. Sepsis-induced immune alterations monitoring by flow cytometry as a promising tool for individualized therapy. *Cytometry B Clin Cytom.* (2016) 90:376–86. 10.1002/cyto.b.21270 26130241

[40] Singhal S Bhojnagarwala P O’Brien S Moon E Garfall A Rao A Origin and role of a subset of tumor-associated neutrophils with antigen-presenting cell features in early-stage human lung cancer. *Cancer Cell.* (2016) 30:120–35. 10.1016/j.ccell.2016.06.001 27374224 PMC4945447

